# Radiographic Evaluation of Spinopelvic Sagittal Alignment Anatomy in Juvenile and Adolescent Idiopathic Scoliosis Patients

**DOI:** 10.3390/tomography12040052

**Published:** 2026-04-07

**Authors:** Ozden Bedre Duygu, Figen Govsa, Anil Murat Ozturk, Gokhan Gokmen

**Affiliations:** 1 Department of Anatomy, Faculty of Medicine, Izmir Bakircay University, 35660 Izmir, Turkey; 2 Digital Imaging and 3D Modelling Laboratory, Department of Anatomy, Faculty of Medicine, Ege University, 35040 Izmir, Turkey; 3 Department of Orthopedics and Traumatology, Faculty of Medicine, Ege University, 35040 Izmir, Turkey; amuratozturk@yahoo.com; 4 Faculty of Medicine, Dokuz Eylul University, 35220 Izmir, Turkey; ggokmen1999@gmail.com

**Keywords:** scoliosis, spinopelvic alignment, sagittal balance, posture, spine

## Abstract

*Background and Objectives*: The association between spinal and pelvic alignment significantly impacts sagittal balance in adults. This study, that is retrospective, aims to investigate sagittal alignment anatomy of the pelvis and spine in juvenile idiopathic scoliosis (JIS) and adolescent idiopathic scoliosis (AIS) patients. *Materials and Methods*: We evaluated nine sagittal parameters from lateral radiographs of 100 JIS and AIS patients, including thoracic kyphosis (TKA), lumbar lordosis (LLA), pelvic tilt (PTA), pelvic incidence (PIA), spinosacral (SSA), sacral slope (SSLA), C7 tilt angles (C7-TA), sagittal vertical axis length (SVAL), and odontoid process hip axis angle (OPHAA) using the ImageJ program. Participants were classified based on their coronal curve group. Analysis of variance compared parameters between curve groups, and Pearson coefficients assessed the relationship between all parameters (*p* < 0.05). *Results*: Female participants had an average age of 13.4, and male participants had an average age of 13.0. Female participants had an average scoliosis degree of 19.3, while male participants had 15.2. PIA, PTA, SSLA, and SSA values were significantly higher in women participants than in men participants (*p* < 0.05). Additionally, PIA, PTA, SSLA, SSA, and OPHAA values were significantly lower in participants with a lower scoliosis degree (*p* < 0.05). We observed a moderately positive association between LLA and TKA, PIA, SSA, and C7-TA. There was also a moderate positive association between spinopelvic alignment parameters and the degree of scoliosis in participants. *Conclusions*: Easily measured values such as PIA, PTA, SSLA, SSA, and OPHAA may be related to severity of vertebral column deformities in patients, making them valuable for monitoring scoliosis patients.

## Simple Summary

Spinopelvic alignment is a critical determinant of sagittal balance and is closely linked to the pathomechanics of scoliosis. This study demonstrated that specific radiographic parameters significantly correlate with curve magnitude and differ by sex in juvenile and adolescent idiopathic scoliosis. Pelvic incidence, pelvic tilt, and sacral slope emerged as robust and easily accessible markers of deformity severity. These parameters provide reliable, clinically applicable tools for risk stratification and objective monitoring of scoliosis progression.

## 1. Introduction

Idiopathic scoliosis is a complex three-dimensional (3D) malalignment of the spine characterized by lateral curvature and vertebral rotation. Although its etiology remains incompletely understood, increasing evidence suggests that sagittal spinal alignment plays a crucial role in the biomechanical stability of the vertebral column and may contribute to the development and progression of spinal deformities [1,2,3,4].

Idiopathic scoliosis is considered to result from disproportionate longitudinal growth between the anterior and posterior columns of the spine, with relative overgrowth of the anterior column. The magnitude of anterior overgrowth is positively associated with the degree of the observable spinal deformity [5]. Insufficient development of the posterior column limits harmonious spinal growth and contributes to structural imbalance. Consequently, the vertebral bodies undergo morphological alterations and axial rotation in order to accommodate spatial and biomechanical constraints. This vertebral rotation leads to a posterior (dorsal) redirection of spinal shear forces [6]. Under normal axial loading conditions, shear forces are distributed in multiple directions; however, in idiopathic scoliosis, these forces become preferentially oriented dorsally. The altered load distribution subjects tissues to atypical mechanical stresses, thereby contributing to increased pain and spinal stiffness.

Mac-Thiong et al. showed that sagittal spinal morphology in idiopathic scoliosis varies according to the apical level of the deformity. Specifically, thoracic scoliosis has been associated with a reduction in thoracic kyphosis, while thoracolumbar and lumbar curves more often retain thoracic kyphosis and are linked to increased upper lumbar lordosis [7].

Idiopathic scoliosis with a predominant thoracic curve is characterized by distinct alterations in sagittal plane alignment, most notably thoracic hypokyphosis. This reduction in physiological kyphosis has been associated with impaired thoracic biomechanics, potentially influencing cosmetic appearance, self-perception, and pulmonary function. Thoracic hypokyphosis may be attributed to the wedge-shaped morphology of the vertebral bodies. In scoliosis, vertebrae are asymmetrically shaped, with greater height on the convex side and anteriorly compared to the concave and posterior aspects [8].

In the 1980s, Dickson et al. advanced a biomechanical theory to account for the familial predisposition associated with idiopathic scoliosis [9]. The maintenance of upright posture depends on a close and functionally integrated relationship between the spine and the pelvis [10]. Legaye et al. and Duval-Beaupère et al. introduced the concept of the pelvic incidence angle (PIA) in 1992 [11,12]. This parameter may be conceptualized as representing the vector of load transmission through the sacral endplate. Pelvic incidence is recognized as the most significant sagittal morphological characteristic of the pelvis [11,12,13].

Pelvic incidence has been defined as an individual specific morphological parameter that is not influenced by the 3D spatial alignment of the pelvis. This anatomical measure remains relatively stable throughout juvenile period; however, it demonstrates a pronounced increase during adolescence and attains its peak value in adulthood. In asymptomatic adults, pelvic incidence exhibits a strong correlation with lumbar lordosis [14,15,16,17]. Moreover, it has been detected as a significant leading to factor in the development of spondylolisthesis [18,19,20].

Roussouly et al. proposed a classification system describing the variability of sagittal spinal alignment in asymptomatic individuals, categorizing the spine into five distinct types. This framework relates the distribution and magnitude of lumbar lordosis to pelvic incidence. Spines with low pelvic incidence (Types 1 and 2) are associated with reduced sacral slope and a relatively short or flattened lumbar lordosis. In Type 1, the lordotic apex is positioned more caudally, whereas in Type 2 it is located more cranially, contributing to a longer but flatter lordotic curve. In contrast, higher pelvic incidence (Types 3 and 4) is linked to increased sacral slope and more pronounced lumbar lordosis, with Type 4 exhibiting the greatest curvature. An intermediate pattern, characterized by a relatively high sacral slope despite low pelvic incidence, has also been described, corresponding to an anteverted pelvic configuration. Overall, sagittal spinal morphology within this classification is primarily determined by sacral slope, pelvic tilt, and the distribution of lordotic segments [21].

Poncet et al. aimed to evaluate a significant association between lumbar lordosis and pelvic incidence in adult patients with scoliosis. Nevertheless, their findings indicated no statistically significant difference in pelvic incidence between adults with scoliosis and healthy control individuals [22].

According to Clement et al., the distal component of lumbar lordosis is closely correlated with pelvic incidence, while the proximal component shows minimal or no dependence on pelvic incidence in idiopathic scoliosis [23].

The pathophysiology of kyphoscoliosis is fundamentally based on its etiological basis. Positional thoracic kyphosis predominantly arises from reversible imbalances in the musculature, which precipitate an abnormal exaggeration of thoracic spinal curvature. Among elderly populations, approximately 60–70% of hyperkyphosis subjects are not attributable to vertebral compression injuries; rather, they are primarily associated with degenerative disc pathology, hereditary predispositions, and atrophy of the spinal extensor musculature. In cases attributable to vertebral fractures, the deformity typically exhibits a characteristic wedge morphology, reflecting a disproportionate loss of anterior vertebral body height relative to the posterior column, thereby contributing to progressive kyphotic deformity. This localized structural compromise exerts secondary biomechanical effects on contiguous spinal segments, ultimately altering normative load distribution and spinal mechanics [24,25].

The association between spine pelvis parameters and pelvic alignment demonstrates notable variability in the literature. Although several studies have reported no significant differences between populations, other investigations have identified an enhanced PIA in individuals with AIS compared to unaffected controls [9,14,26,27,28,29].

With respect to scoliosis concordance among twins, the literature includes data in which zygosity—either monozygotic or dizygotic—was verified for all participants. Twenty-seven twin pairs demonstrated concordance for scoliosis among 37 monozygotic twin pairs, whereas only 11 of 31 dizygotic twin pairs were concordant. This disparity in concordance rates between monozygotic and dizygotic twins was statistically significant, highlighting the potential influence of genetic factors in the etiology of AIS [30].

Numerous investigations have addressed spinal stability in adolescent idiopathic scoliosis (AIS); nevertheless, the correlation between spinal alignment and pelvic morphology remains insufficiently defined in the existing literature [1,2,3,4,5,6]. Therefore, the present study aimed to evaluate sagittal orientation of the spine and pelvis in patients with JIS and AIS. Additionally, the study aimed to examine the potential interaction between sagittal orientation and the specific pattern of the scoliotic curve. Vertebral bodies are observed to rotate further from the central line than the posterior components of the spine, largely due to the constraining effect of the posterior ligamentous structures. This rotational phenomenon gives rise to apical lordosis and produces an apparent lateral displacement on anterior–posterior radiographic imaging [1,3,31,32].

Biomechanical analyses have demonstrated that rotational stability is influenced, in part, by the degree of posteriorly directed shear forces [29,33]. These forces are contingent upon the spatial alignment of the vertebrae within the sagittal plane. Accordingly, the length and inclination of posteriorly inclined spinal portions are critical factors in assessing the rotational rigidity of the spine [10,34,35,36].

If sagittal spinal alignment and morphology influence in the etiopathogenesis of AIS by modulating the rotational stability of specific spinal segments, and if these morphological characteristics possess a heritable component, it is plausible that parents of affected individuals may exhibit distinctive sagittal spinal profiles. We postulated that variations in spinopelvic parameters—including pelvic incidence (PIA), pelvic tilt (PTA), and sacral slope angles (SSLA)—may reflect inherent biomechanical and structural differences in the pelvis and spine among scoliosis patients. Such alterations could be involved in both the initiation and development of scoliotic deformity, thereby influencing overall sagittal balance. The primary objective of the present study was to assess the correlation between spinal deformity, pelvic alignment, and sagittal balance in the context of scoliosis.

In addition, we aimed to examine whether scoliosis severity, as measured by the Cobb angle, correlates with discrete modifications in sagittal spinal alignment and spinopelvic parameters. Understanding these associations may provide insight into the compensatory—or maladaptive—mechanisms of spinal and pelvic morphology in response to scoliotic curvature. Accordingly, this study aimed to comprehensively evaluate sagittal spinal profiles and spinopelvic alignment in JIS and AIS patients.

## 2. Materials and Methods

### 2.1. Subjects

#### Juvenile and Adolescent Idiopathic Scoliosis Group

Radiographs from a consecutively selected cohort of 100 patients (33 boys and 67 girls) diagnosed with JIS or AIS at the authors’ scoliosis clinic were retrospectively analyzed within an Anatolian population. Researchers were used for a systematic sampling method with a sample interval of 2.

Eligibility criteria contained a Cobb angle higher than 10°, age between 5 and 20 years, absence of neurological deficits, no history of prior spinal surgery, and absence of spondylolisthesis. All patients underwent standardized digital standing lateral and posteroanterior radiographic images of the spinopelvic region as part of their routine clinical assessment.

**Figure 1:**
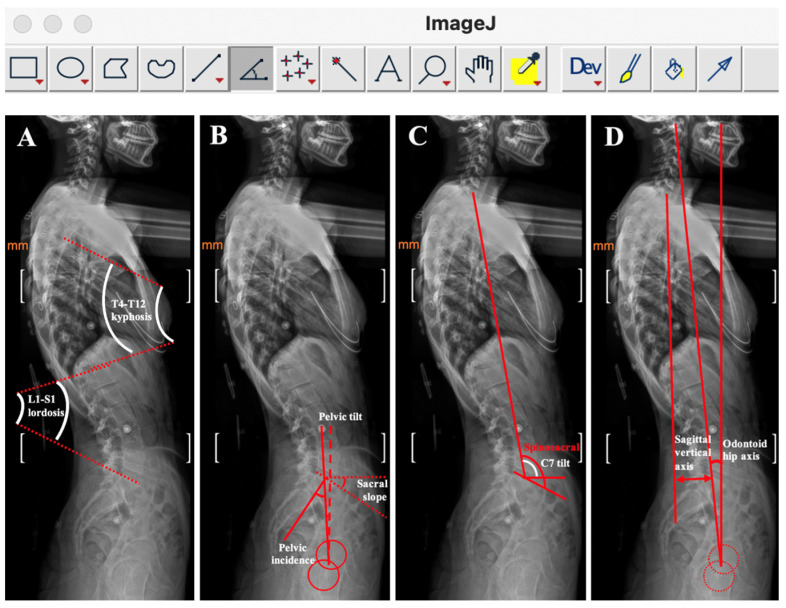
Sagittal alignment parameters. (**A**). Measurement of the thoracic kyphosis angle and the lumbar lordosis angle. (**B**). Measurement of the pelvic incidence angle, the pelvic tilt angle and the sacral slope angle. (**C**). Measurement of the spinosacral angle and C7 tilt angle. (**D**). Sagittal balance parameters; measurement of the length of the sagittal vertical axis and the dens axis hip axis angle.

**Figure 2:**
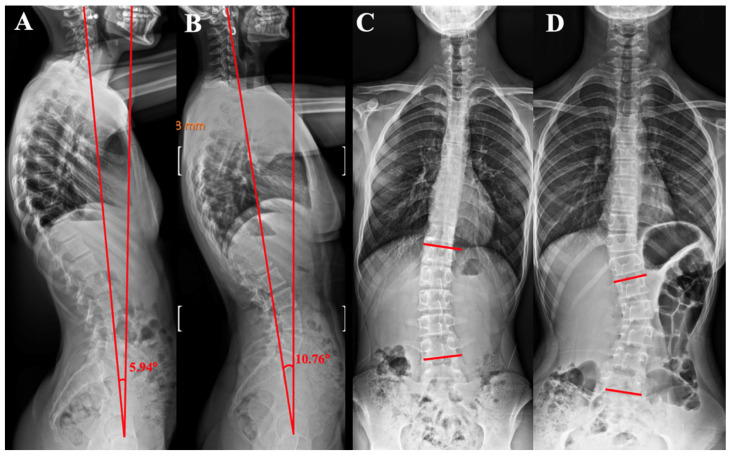
(**A**). Measurement of the dens axis hip axis angle in a scoliosis patient with an 11 degree lumbar curvature. (**B**). Measurement of the dens axis hip axis angle in a scoliosis patient with a 26 degree lumbar curvature. (**C**). Measurement of the Cobb angle in a scoliosis patient with an 11 degree lumbar curvature. (**D**). Measurement of the Cobb angle in a scoliosis patient with a 26 degree lumbar curvature.

Sagittal spinal and pelvic alignment was independently evaluated by two observers utilizing ImageJ 1.48v (developed by the National Institutes of Health, Bethesda, MD, USA). Each observer performed three repeated measurements, and the mean values were subsequently computed to ensure reliability. The software required interactive digitization of predefined anatomical landmarks on lateral radiographs, including the anterior vertebral body walls from T4 to L5, the sacral endplate, and the contours of the femoral heads (Table 1, Table 2 and Table 3). Nine parameters in the sagittal plane were subsequently derived and analyzed to characterize spinopelvic morphology (Figure 1 and Figure 2).

***Thoracic Kyphosis (TKA):*** This angle that is measured using the Cobb method is defined between the upper border of the T4 vertebra and the lower border of the T12 vertebra (Figure 1A).

***Lumbar Lordosis (LLA):*** This angle that is determined via the Cobb method is formed between the upper border of the L1 vertebra and the lower border of the L5 vertebra (Figure 1A).

**Figure 3:**
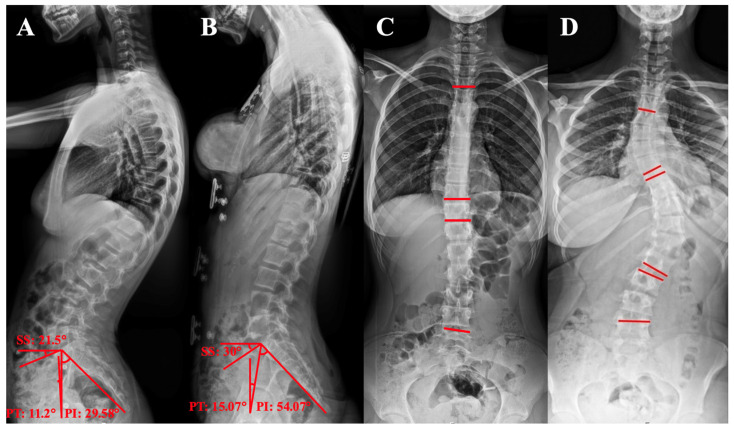
(**A**). Measurement of pelvic incidence (**PI**), pelvic tilt (**PT**), and sacral slope (**SS**) angles in a scoliosis patient with 11 degree thoracic and 11 degree lumbar curvatures. (**B**). Measurement of pelvic incidence, pelvic tilt, and sacral slope angles in a scoliosis patient with 27 degree thoracic and 24 degree lumbar curvatures. (**C**). Measurement of the Cobb angle in a scoliosis patient with 11 degree thoracic and 11 degree lumbar curvatures. (**D**). Measurement of the Cobb angle in a scoliosis patient with 27 degree thoracic and 24 degree lumbar curvatures.

***Pelvic Incidence (PIA):*** The angle is defined as the angle between a line perpendicular to the sacral upper border and a line connecting the center of the sacral upper border to the hip axis (Figure 1B and Figure 3A,B).

***Sacral Slope (SSLA):*** The angle formed between the sacral upper border and a horizontal reference line on the lateral radiograph (Figure 1B and Figure 3A,B).

***Pelvic Tilt (PTA):*** The angle is defined as the angle between a vertical reference line and the line connecting the center of the superior sacral endplate to the hip axis. A positive value denotes that the hip axis lies anterior to the sacral endplate center (Figure 1B and Figure 3A,B).

***Spinopelvic Angle (SPA):*** The angle formed between the vertebral axis and the pelvic axis as observed on lateral radiography (Figure 1C).

***C7 Vertebra Identification:*** The C7 vertebra is located at the cervical thoracic junction. A vertical axis is drawn from the posterior superior aspect of C7 to serve as a reference.

***Horizontal Reference Line:*** A horizontal line is established on the lateral radiograph, typically aligned parallel to the lower edge of the image.

***C7 Line:*** A line is drawn along the posterior surface of C7 from its superior edge; this line is not necessarily parallel to the horizontal reference line.

***C7 Tilt (C7-TA):*** The angle is between the posterior axis of C7 and the horizontal reference line, as visualized on a lateral radiograph (Figure 1C).

***Posterior Superior Iliac Spine (PSIS) Point:*** The PSIS, located at the posterior superior aspect of the pelvis, is identified and marked for reference.

***Vertical Reference Line:*** A line is derived from the superior aspect of C7 to the PSIS point, serving as a sagittal alignment reference.

***Sagittal Vertical Axis (SVAL):*** This length is described as the horizontal length between the vertical reference line from C7 and the PSIS point (Figure 1D).

***Sagittal Vertical Axis Length (SVEU):*** This is assessed as the length between the vertical line from C7 and the posterior superior aspect of the S1 vertebra.

***Dens Axis–Hip Axis Angle (DA-HA):*** This is formed between two reference lines: one extending vertically from the tip of the dens (odontoid process) of C2, and the other extending from the hip axis upward toward the cervical spine. The hip axis is described as the midpoint between the centers of the right and left femoral heads (Figure 1D and Figure 2A,B).

***Geometric Relationship:*** The pelvic incidence angle (PIA) is mathematically equivalent to the total of the sacral slope angle (SSLA) and the pelvic tilt angle (PTA). The hip axis is determined by software based on femoral head identification, accounting for potential distortions arising from radiographic beam divergence, magnification, or pelvic asymmetry.

### 2.2. Radiographic Acquisition Protocol

Standardized full-spine scoliosis radiographs were obtained, encompassing the anatomical region from the cranium to both femoral heads within a single exposure. The cervical spine was positioned in the upper field of the image, while the pelvis was included in the lower field to ensure complete spinopelvic assessment.

All images were acquired with a fixed source-to-image distance of 180 cm (72 inches) to minimize magnification variability and ensure measurement reliability. Patients were positioned in an upright standing posture, with the knees fully extended and the feet placed at shoulder-width apart to maintain a standardized and reproducible stance.

For lateral projections, subjects were instructed to maintain a neutral forward gaze in order to avoid cervical flexion or extension. The upper extremities were positioned with the elbows flexed and the hands resting over the clavicular region, minimizing superimposition artifacts over the thoracic spine.

### 2.3. Measurement Method

Spinopelvic parameters were quantified on sagittal radiographic images using a custom-developed, semi-automated software platform. Vertebral segmentation from C5 through L5 was performed by identifying the four corner landmarks of each vertebral body, whereas the superior endplate of S1 was delineated via the anterior superior and posterior superior corner points. The femoral heads were represented by circles fitted using a three-point selection method (Figure 1B,D).

All measurements were conducted by two experienced anatomists. Observer FG performed a single measurement session, while observer OB completed two independent measurement sessions. To minimize bias, all radiographs were anonymized and randomized prior to evaluation. Intra- and inter-observer reliability for each parameter was evaluated using intraclass correlation coefficients (ICC).

This study was conducted and completed over a 12-month period.

### 2.4. Ethics

Ethical approval was obtained from the Non-interventional Clinical Researches Ethics Committee of lzmir Bakırçay University (Decision No: 1542; Research No: 1522; 17 April 2024). All procedures were carried out in accordance with the ethical rules and the principles of the Declaration of Helsinki. Permission was obtained from participants and copyright holders.

### 2.5. Statistical Analysis

All data were processed using IBM SPSS Statistics, version 23 (IBM Corp., Armonk, NY, USA). Pelvic incidence angle (PIA) values were compared with historical normative data using two-tailed Student’s *t* tests (Table 1, Table 2 and Table 3). Associations among all measured spinopelvic and spinal parameters were analyzed using Pearson correlation coefficients (Table 4). A significance level of *p* = 0.05 was applied for all statistical tests.

## 3. Results

A total of 100 patients with scoliosis were included in the study, comprising 33 males and 67 females, corresponding to a male-to-female ratio of 1:2. According to the Scoliosis Research Society (SRS) classification, patients were categorized as having juvenile or adolescent idiopathic scoliosis. The cohort consisted of 21 patients aged 4–10 years and 79 patients aged 11–20 years.

Coronal curve patterns were categorized into four groups (thoracic, thoracolumbar, lumbar, and double major) based on curve distribution, using a simplified classification adapted from the Lenke system, as the primary focus of the study was sagittal alignment. Regarding curve distribution, lumbar scoliosis was the most prevalent type, observed in 45% of patients (n = 45; 31 females, 14 males), followed by double major scoliosis in 25% (n = 25; 17 females, 8 males), thoracic scoliosis in 20% (n = 20; 13 females, 7 males), and thoracolumbar scoliosis in 10% (n = 10; 6 females, 4 males).

For the overall cohort, descriptive statistics, including means and standard deviations, along with the results of the analysis of variance (ANOVA), are presented in Table 1, Table 2 and Table 3. Statistically significant sex-related differences were identified for pelvic tilt (PTA), pelvic incidence (PIA), spinopelvic (SPA), and sacral slope angles (SSLA) (Table 1). Age-related significant differences were detected for lumbar lordosis angle (LLA), pelvic tilt angle (PTA), and sacral slope angles (SSLA) (Table 2). Post hoc comparisons indicated that patients with Cobb angles between 21° and 40° demonstrated significantly greater values of PIA (*p* < 0.001), PTA (*p* < 0.001), SSLA (*p* < 0.001), SPA (*p* < 0.001), and sagittal vertical axis length (SVEU) (*p* < 0.02) compared with those presenting with Cobb angles between 11° and 20°. Conversely, thoracic kyphosis (TKA), lumbar lordosis (LLA), and C7 tilt angles (C7-TA) did not exhibit statistically significant differences according to sex or Cobb angle stratification (Table 1 and Table 3).

Across the study groups, pelvic parameters—namely sacral slope (SSLA), pelvic incidence (PIA), and pelvic tilt angle (PTA)—remained largely consistent, with no meaningful differences observed (Table 1, Table 2 and Table 3). The outcomes of the Pearson correlation analyses assessing associations among sagittal alignment variables for the overall cohort are presented in Table 4.

Thoracic kyphosis (TKA) and lumbar lordosis angles (LLA) displayed generally weak relationships with the other sagittal alignment variables, and these associations were not statistically significant. Pelvic incidence (PIA) and pelvic tilt angles (PTA) showed low-grade correlations with the C7 tilt angle (C7-TA). Spinopelvic angle (SPA) was consistently correlated with sacral slope (SSLA) and pelvic incidence angle (PIA) in all groups, with the strongest associations observed in patients whose Cobb angles ranged between 21° and 40° (Table 3).

## 4. Discussion

**Figure 4:**
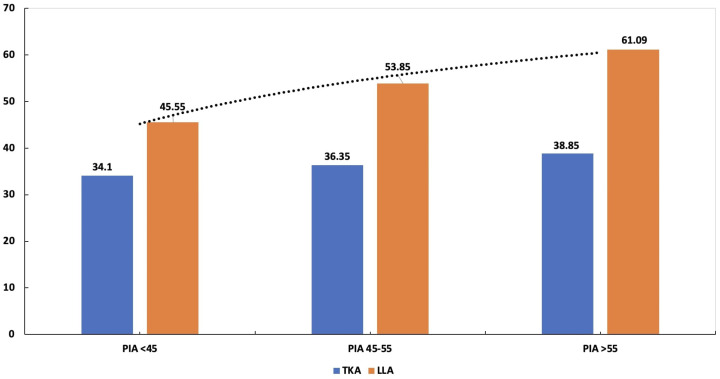
Graphic bar chart displaying the values of thoracic kyphosis angle (TKA) and lumbar lordosis angle (LLA) across three groups based on pelvic incidence angle (PIA): PIA < 45, PIA 45–55, and PIA > 55.

The objective of the present investigation was to analyze sagittal orientation of the spinopelvic region in patients with juvenile JIS and AIS. The evaluation included thoracic kyphosis (TKA), lumbar lordosis (LLA), pelvic tilt (PTA), pelvic incidence (PIA), sagittal spinopelvic (SSA), sacral slope (SSLA), C7 tilt angles (C7-TA), sagittal vertical axis length (SVAL), and the odontoid process–hip axis angle (OPHAA) (Figure 1, Figure 2, Figure 3 and Figure 4; Table 1, Table 2, Table 3 and Table 4). These radiographic parameters are commonly applied in the assessment of spinal pathologies and postural characteristics. Together, they provide insight into global sagittal balance, the biomechanical interplay between the pelvis and spinal column, and potential structural abnormalities.

C7-TA and SVAL are alignment measures that may vary according to patient positioning during image acquisition. In contrast, the dens–hip axis parameter is not directly dependent on positional variation; rather, it reflects a compensated alignment within the sagittal plane. Accordingly, C7-TA, SVAL, and the dens–hip axis angle can be interpreted as indicators of compensatory mechanisms influenced by the magnitude of spinal deformity and underlying postural disturbances. These angular measurements are instrumental in elucidating the adaptive responses of the spine and lower extremities (Figure 1, Figure 2, Figure 3 and Figure 4). Their relevance is particularly evident in comprehensive evaluations of global spinal alignment, and they constitute important tools in both diagnostic assessment and therapeutic planning within the fields of spinal surgery, orthopedics, and rehabilitation.

Radiographic protocols for the evaluation of scoliosis may vary between radiology departments. In patients presenting with a leg-length discrepancy, a compensatory block should be placed beneath the shorter limb—or beneath the convex side of the spinal curvature—prior to image acquisition. Standard imaging projections typically include erect posteroanterior (PA) or anteroposterior (AP) views, erect lateral views, and additional lateral bending PA/AP views. Precise attention to radiographic technique is critical, as minor alterations in magnification, rotation, or patient positioning can significantly affect the measured values of spinal curvature. Consistent adherence to a standardized imaging protocol helps minimize these potential sources of error. Scoliosis radiographs should capture the cervical spine at the superior aspect and the pelvis at the inferior aspect of the image. For assessments of sagittal stability, it is particularly crucial to comprise the cranium through to both femoral heads within the same radiographic image. The patient is positioned approximately 72 inches from the radiation source, with knees fully stretched and feet placed shoulder-width apart. During lateral imaging, the patient is instructed to look straight ahead while placing the hands over the clavicles with elbows flexed, thereby preventing the upper extremities from overlapping the spinal column. Pelvic and breast shields may be applied to decrease radiation exposure, and a compensating filter is utilized to ensure uniform visualization of bone density throughout the spine [37,38,39].

Adolescents in this study were categorized solely according to their coronal curve patterns, as the primary focus was the evaluation of sagittal orientation. The observed values for thoracic kyphosis (TKA), lumbar lordosis angle (LLA), and pelvic parameters (PIA, SSLA, and PTA) were consistent with ranges reported in the existing literature for AIS [1,4,9,29,34]. TKA was significantly reduced in patients exhibiting a thoracic curve component compared to those with a lumbar curve, in agreement with previous studies examining sagittal alignment across different scoliotic curve types [4,40,41,42]. Normal TKA is generally defined as 10–20°, although reference ranges of 21–40° have also been proposed. It should be noted that TKA measurement using the Cobb technique on lateral radiographic images may underestimate kyphosis loss in patients with thoracic curves, as vertebral rotation and wedging can result in an apparently reduced angle when viewed from a true lateral perspective at the curve apex. Multiple studies have demonstrated that TKA is typically smaller in thoracic curves relative to control spines [4,29,35,43], a phenomenon attributed to abnormal growth patterns of thoracic vertebral bodies in AIS. Conversely, some reports have found no significant differences in TKA between scoliosis patients and controls [4,29,35,44]. For Cobb angle measurements of thoracic kyphosis, the upper border of T1 and the lower border of T12 were used as reference points. In instances where T1 was obscured by soft tissue, the next highest visible vertebra was employed [45]. Previous studies, however, did not focus exclusively on AIS and often lacked specification of curve patterns within their cohorts. A major strength of the present study is the simultaneous assessment of TKA, LLA, and pelvic parameters, providing a comprehensive overview of sagittal and spinopelvic alignment (Table 1, Table 2, Table 3 and Table 4). To ensure standardization, thoracic kyphosis was measured between T4 and T12 in all patients, as only the superior endplate of T4 was consistently visible. Consequently, the T4–T12 Cobb measurement may not fully represent the global thoracic curvature and can differ from T1–T12 measurements depending on segmental alignment. In contrast, prior investigations frequently limited their analyses to the cervical, lumbar, or thoracolumbar regions, resulting in incomplete evaluations of sagittal spinal orientation.

The results of this study regarding TKA align with previous research demonstrating an association between reduced kyphosis and thoracic AIS. Although TKA values in scoliotic curves generally approximated normal ranges, reductions were most pronounced in the thoracic region, suggesting that TKA is largely determined by the morphology and orientation of intervertebral discs and vertebral bodies, both of which are altered in AIS. The absence of a significant correlation between TKA and LLA, except in cases involving thoracolumbar curves, further supports this interpretation. Consistent with these findings, most studies report no direct association between TKA and LLA in adolescents without spinal deformity, whereas a significant relationship has been observed in adults [14,16,44,45]. Nonetheless, numerous investigations have documented a significant correlation between TKA and LLA in both healthy individuals and those with scoliosis [3,35,36,41,42,46,47]. Therefore, in the evaluation of the developing adolescent spine, the influence of vertebral and disc morphology on TKA should be carefully considered when interpreting standing sagittal alignment.

For most scoliotic curve types, TKA did not exhibit significant correlations with any pelvic parameters, which is consistent with the observed lack of association between TKA and LLA and reflects the relative independence of the thoracic spine from pelvic morphology. Although LLA tended to be greater in patients with lumbar curves, this difference did not reach statistical significance. Moreover, LLA values in lumbar curves were comparable to those described in healthy subjects. Previous studies, such as Rubery et al., reported significantly increased LLA in patients with scoliosis compared to controls; however, they did not specify the curvature classification or scoliosis etiology [33]. In the present study, LLA values across all curve types remained within established normal ranges.

Pelvic geometry demonstrated a notable influence on LLA, with strong correlations observed between LLA and both PIA and SSLA across all groups. This relationship is essential for the maintenance of sagittal stability in both physiologic and pathologic adult spines, and similar associations between SSLA and LLA have been documented in healthy adolescents.

The compensatory mechanisms employed by AIS patients to maintain sagittal balance, despite the absence of correlations between certain parameters—particularly TKA and LLA—cannot be fully elucidated by the present findings. While this study focused on the pelvis, thoracic spine, and lumbar spine, sagittal stability regulation also involves active adaptation from the lower extremities (Table 1, Table 2, Table 3 and Table 4). Furthermore, TKA and LLA were measured using the Cobb technique between fixed vertebral levels; assessing global TKA and LLA using inflection points may provide a more comprehensive evaluation of sagittal plane alignment (Figure 4). Additionally, incorporating assessments of the global center of gravity via force plate analysis could further complement the evaluation of sagittal radiographic parameters.

Farshad et al. analyzed spinopelvic parameters across various Lenke curvature classifications in AIS and reported that overall spinopelvic balance did not differ significantly among the curve classifications. Minor alterations in spinopelvic alignment were observed specifically in Lenke types 5 and 6, which involve primary curves in the thoracolumbar and lumbar regions, with mean values of pelvic incidence (PIA) 44°, sacral slope (SSLA) 34°, and pelvic tilt angles (PTA) 10° when compared to normative measurements. In the context of AIS surgical management, several studies have documented changes in spinopelvic parameters postoperatively. La Maida et al. observed a statistically significant rise in PTA following posterior spinal instrumentation and fusion, while Tanguay et al. identified significant correlations between lumbar lordosis and pelvic parameters within and below the fusion levels in a cohort of 60 AIS patients undergoing similar surgical intervention [9,27,28,29].

Following the seminal work of Duval-Beaupère et al. on the correlation between pelvic morphology and spinal alignment, numerous studies have investigated sagittal spinopelvic parameters in adults. However, relatively few studies have focused on this relationship in participants with AIS, and the existing literature provides limited detail regarding the association between spinal balance and pelvic configuration in this population [7,8,9,10,11,12,13,14,15,16,17,18,19,20,21,22,23,24,25,26,27,28,29,30,31,32,33,34,35,36,37,38,39,40,41,42,43,44,45,46,47,48].

Although pelvic incidence angle (PIA) has been reported to be higher in AIS patients compared to historical adolescent controls, it is important to note that sagittal pelvic morphology may not directly drive the pathogenesis of AIS [47,49,50]. This is supported by the lack of a significant interaction between pelvic parameters and curve type, particularly in thoracic curves, where no association between PIA and TKA was observed despite documented reductions in TKA in early thoracic AIS. Additionally, an increased PIA is theoretically associated with a higher LLA, which may serve as a protective mechanism against the development of a straight spine by reducing the risk of coronal plane buckling [42,43,44,45,46,49,50,51]. Sagittal curvatures are thus widely recognized as a safeguard against coronal deformity initiation.

It is plausible that suboptimal sagittal pelvic structure could influence the mechanical loads imposed on the spine and thereby influence AIS progression [52,53]. Conversely, an elevated PIA may reflect a compensatory adaptation aimed at increasing LLA, particularly in thoracic AIS, where TKA is often reduced. In the present study, the absence of a correlation between PIA and Cobb angle does not exclude the possibility of a relationship between sagittal pelvic structure and curve progression. It is crucial to recognize that, as a cross-sectional analysis, this study cannot capture temporal changes in spinal or pelvic parameters, limiting conclusions regarding causality between PIA and the Cobb angle [23,40]. Longitudinal studies including both AIS patients and healthy adolescents are required to clarify the role of pelvic morphology in the pathogenesis of AIS. Moreover, given the three-dimensional nature of AIS, incorporating three-dimensional spinal parameters would provide a more extensive understanding of the relationship between sagittal pelvic orientation and scoliosis.

The Multi-Ethnic Alignment Normative Study (MEANS) conducted by Serdar et al. studied skeletal orientation—comprising the spine and lower extremities—in the largest cohort of asymptomatic adults to date [16]. This prospective study enrolled individuals aged 18–80 years from multiple centers in France, Japan, Singapore, Tunisia, and the United States. The study suggested that TKA is influenced not only by biomechanical changes occurring within the spine and lower extremities with age but also by environmental factors, cultural postural habits, and lifestyle choices. PIA and TKA were hypothesized to be key determinants in establishing the level of lumbar lordosis necessary to maintain upright posture and sagittal balance, ensuring proper alignment of the center of gravity.

Findings indicated stronger correlations between LLA, PIA, and TKA in younger individuals compared to older cohorts, reflecting a diminished capacity of LLA to compensate for age-related increases in TKA [16]. Key observations included a relatively stable LLA across age groups, a significant increase in TKA in individuals over 50 years, and a concomitant rise in SVAL, with LLA showing minimal variation. To maintain alignment of the odontoid process over the knees in the context of increased TKA and SVAL, participants adopted compensatory mechanisms such as increased cervical lordosis, enhanced hip extension, elevated PTA, and greater knee flexion [16].

The elevated PIA observed in this study may serve as a secondary marker of fundamental processes contributing to adolescent idiopathic scoliosis (AIS) (Figure 4). In typically developing individuals, PIA gradually increases from the onset of independent walking and continues to develop into adulthood. The higher PIA, PTA, SSLA, and SPA observed in AIS patients (Table 3) may reflect altered skeletal growth patterns, a phenomenon commonly reported in AIS. Girls with AIS frequently present with taller and leaner body habitus compared to controls, and increased stature has been associated with a less favorable prognosis in scoliosis.

We hypothesized that variations in spinopelvic parameters—including PIA, PTA, and SSLA—may reflect differences in the biomechanical and structural characteristics of the spinopelvic region in scoliosis patients. Such alterations could contribute to the development and progression of scoliotic curves, thereby influencing overall sagittal balance. Accordingly, this study intended to elucidate the correlation between spinal deformity, pelvic alignment, and sagittal balance in AIS. In addition, we sought to examine whether scoliosis severity, quantified by Cobb angle, correlates with specific changes in sagittal spinal alignment and spinopelvic parameters. Understanding these relationships may provide insight into how spinal and pelvic anatomy adapts—or fails to compensate—as the scoliotic curve progresses, thereby enhancing comprehension of the pathophysiology of scoliosis and its impact on sagittal alignment.

These findings may have relevant implications for the clinical management of JIS and AIS. The observed variability in spinopelvic parameters, particularly pelvic incidence, pelvic tilt, and sacral slope, suggests that evaluation of sagittal alignment should extend beyond coronal deformity assessment. Consideration of patient-specific pelvic morphology may contribute to a more comprehensive understanding of sagittal balance. Furthermore, the association between pelvic parameters and curve magnitude indicates that these variables could play a role in guiding alignment targets and treatment strategies. The relative preservation of thoracic kyphosis and lumbar lordosis across certain groups may reflect underlying compensatory mechanisms, highlighting the importance of avoiding excessive correction that could disrupt physiological balance. Overall, incorporating spinopelvic parameters into routine assessment may support more individualized decision-making and provide additional insight into alignment patterns in JIS and AIS.

The observed differences in PIA between AIS patients in this study and historical adolescent cohorts may also reflect the limitations inherent in using retrospective comparisons. One notable limitation is the absence of MRI evaluation, precluding the exclusion of underlying conditions such as Chiari malformations or syringomyelia. One limitation of this study is the heterogeneity in age across the groups, which may have influenced the results. Furthermore, a contemporary control group of juvenile and adolescents without spinal deformity was not included; consequently, pelvic parameters were compared with values reported in historical studies. Future research directly comparing AIS patients with contemporaneously assessed healthy adolescents, using standardized measurement techniques, is necessary to validate potential differences in pelvic morphology. A normative database of adolescents without spinal deformity is currently under development to facilitate such comparisons.

## 5. Conclusions

Spinopelvic parameters vary significantly with sex, age, and curve magnitude in juvenile and adolescent scoliosis, with pelvic incidence, pelvic tilt, sacral slope, and spinopelvic angle emerging as the most discriminative factors. In contrast, thoracic kyphosis and lumbar lordosis appear relatively preserved, suggesting adaptive mechanisms that maintain global sagittal alignment. The strong interdependence among pelvic parameters, particularly the association of sacral slope with overall spinopelvic alignment, underscores the pivotal role of pelvic morphology in deformity progression. Clinically, these findings highlight that accurate assessment of pelvic parameters is essential for a comprehensive understanding of sagittal balance and should be systematically integrated into evaluation and treatment planning to optimize patient-specific management. Future research should focus on the role of PIA in the pathogenesis and progression of JIS and AIS through longitudinal studies involving both affected patients and healthy adolescent controls.

## Figures and Tables

**Table 1:** Scoliosis in juvenile and adolescents: comparative table of length and angle values by gender.

Measurements/Variables	Girls	Boys	*p*-Value
**Thoracic Kyphosis Angle (TKA)**	36.3 ± 6.0°	35.8 ± 4.9°	0.6
**Lumbar Lordosis Angle (LLA)**	52.7 ± 12.8°	52.1 ± 10.9°	0.5
**Pelvic Incidence Angle (PIA)**	52.0 ± 10.6°	46.3 ± 7.4°	**0.01**
**Pelvic Tilt Angle (PTA)**	11.3 ± 6.5°	8.4 ± 4.2°	**0.03**
**Sacral Slope Angle (SSLA)**	32.2 ± 12.5°	25.4 ± 8.1°	**0.002**
**Spinopelvic Angle (SPA)**	134.9 ± 12.8°	128.4 ± 11.4°	**0.02**
**C7 Tilt Angle (C7-TA)**	95.9 ± 4.3°	95.2 ± 4.4°	0.4
**Sagittal Vertical Axis Length (SVEU)**	31.3 ± 16.9 mm	29.9 ± 17.2 mm	0.7
**Dens Axis Hip Axis Angle (DAHA)**	5.9 ± 3.2°	5.2 ± 2.5°	0.3

***Note:* Values are presented as mean and standard deviation. Paired *t* tests then were used to examine any differences between girls’ and boys’ mean values. Bold results showed significant differences between girls’ and boys’ mean values (*p* < 0.05).**

**Table 2:** Scoliosis in juvenile and adolescents: comparative table of length and angle values by age group.

Measurements/Variables	Age Group (Years)		
	4–10	11–20	*p*-Value
**Thoracic Kyphosis Angle (TKA)**	35.8 ± 6.41°	36.52 ± 5.76°	0.62
**Lumbar Lordosis Angle (LLA)**	48.27 ± 12.05°	54.79 ± 12.31°	**0.03**
**Pelvic Incidence Angle (PIA)**	49.94 ± 10.98°	51.1 ± 10.19°	0.64
**Pelvic Tilt Angle (PTA)**	13.58 ± 7.65°	10.01 ± 5.64°	**0.01**
**Sacral Slope Angle (SSLA)**	35.65 ± 15.86°	29.26 ± 10.24°	**0.02**
**Spinopelvic Angle (SPA)**	135.73 ± 14.34°	132.89 ± 12.3°	0.36
**C7 Tilt Angle (C7-TA)**	94.91 ± 5.44°	96.01 ± 3.99°	0.3
**Sagittal Vertical Axis Length (SVEU)**	30.74 ± 17.44 mm	31.02 ± 16.89 mm	0.94
**Dens Axis Hip Axis Angle (DAHA)**	6.75 ± 4.47°	5.57 ± 2.52°	0.11

***Note*: Values are presented as mean and standard deviation. Paired *t* tests then were used to examine any differences between age groups’ mean values. Bold results showed significant differences between age groups’ mean values (*p* < 0.05).**

**Table 3:** Scoliosis in juvenile and adolescents: length and angle values based on Cobb’s angle.

Measurements/Variables	Cobb Angle		*p*-Value
	11–20°	21–40°	
**Thoracic Kyphosis Angle (TKA)**	35.9 ± 5.5°	36.7 ± 6.1°	0.2
**Lumbar Lordosis Angle (LLA)**	52.6 ± 12.4°	53.3 ± 11.4°	0.4
**Pelvic Incidence Angle (PIA)**	47.3 ± 9.6°	56.3 ± 8.4°	**<0.001**
**Pelvic Tilt Angle (PTA)**	8.7 ± 5.6°	13.9 ± 5.7°	**<0.001**
**Sacral Slope Angle (SSLA)**	25.9 ± 9.2°	38.6 ± 12.0°	**<0.001**
**Spinopelvic Angle (SPA)**	129.4 ± 12.2°	140.2 ± 10.7°	**<0.001**
**C7 Tilt Angle (C7-TA)**	31.6 ± 16.9 mm	29.7 ± 17.0 mm	0.6
**Sagittal Vertical Axis Length (SVEU)**	5.3 ± 2.5°	6.6 ± 3.7°	**0.02**

***Note*: Values are presented as mean and standard deviation. Paired *t* tests then were used to examine any differences between Cobb angles. Bold results showed significant differences between Cobb angle values (*p* < 0.05).**

**Table 4:** Pearson correlation analysis of length and angle values in juvenile and adolescents with scoliosis: a comprehensive overview of all parameters.

	TKA	LLA	PIA	PTA	SSLA	SPA	C7-TA	SVEU	DAHA	Age Group	Cobb Angle
**TKA**	1	0.327	0.206	0.164	0.105	0.190	0.238	0.120	0.149	0.074	0.108
**LLA**		1	0.448	0.139	0.287	0.527	0.301	0.236	0.085	0.227	0.081
**PIA**			1	0.615	0.629	0.566	−0.191	0.148	0.139	0.129	0.446
**PTA**				1	0.401	0.237	−0.138	0.184	0.218	−0.145	0.424
**SSLA**					1	0.811	−0.008	0.066	0.297	−0.076	0.517
**SPA**						1	0.240	0.194	0.304	0.033	0.424
**C7-TA**							1	0.514	0.500	0.132	−0.047
**SVEU**								1	0.444	−0.020	−0.053
**DAHA**									1	−0.151	0.230
**Age group**										1	0.060

## Data Availability

The datasets analyzed during the current study are not publicly available due to ethical and institutional restrictions.

## Abbreviations

The following abbreviations are used in this manuscript:

JIS	Juvenile Idiopathic Scoliosis
AIS	Adolescent Idiopathic Scoliosis
TKA	Thoracic Kyphosis Angle
LLA	Lumbar Lordosis Angle
PIA	Pelvic Incidence Angle
PTA	Pelvic Tilt Angle
SSLA	Sacral Slope Angle
SSA	Spinosacral Angle
SPA	Spinopelvic Angle
C7-TA	C7 Tilt Angle
SVAL	Sagittal Vertical Axis Length
OPHAA	Odontoid Process Hip Axis Angle
